# Cytochrome bd-II oxidase CyxA promotes the pathogenicity of *Klebsiella pneumoniae* by resisting oxidative stress

**DOI:** 10.1080/21505594.2025.2590244

**Published:** 2025-11-14

**Authors:** Xiao Xiao, Guoyuan Song, Huigai Lu, Wen Zheng, Panpan Meng, Wei Peng, Jing Yang, Miao Wang, Jianyong Zhu, Jiao Wang, Bei Li, Moran Li

**Affiliations:** a Department of Respiratory, Renmin Hospital, School of Basic Medical Sciences, Hubei University of Medicine, Shiyan, China; b Shiyan Key Laboratory of Virology, Hubei University of Medicine, Shiyan, China; c Out-Patient Department, Taihe Hospital, Hubei University of Medicine, Shiyan, China; d School of Basic Medicine, Hubei University of Arts and Science, Xiangyang, China; e Biomedical Research Institute, Hubei University of Medicine, Shiyan, China

**Keywords:** *Klebsiella pneumoniae*, oxidative stress, cytochrome bd oxidase, CyxA, pathogenicity

## Abstract

Adaptation to oxidative stress is crucial for survival of *Klebsiella pneumoniae* in external environments and within infected hosts. Cytochrome bd oxidase contributes to oxidative stress resistance and enhances the pathogenicity of several pathogens. In this study, we explored the role of cytochrome bd-II oxidase CyxA in *K. pneumoniae*’s response to oxidative stress and its overall pathogenicity. The expression level of *cyxA* was significantly increased in response to oxidative stress in the wild-type strain (WT). Deletion of *cyxA* reduced *K. pneumoniae*’s resistance to exogenous hydrogen peroxide (H_2_O_2_) and nitric oxide (NO). Additionally, the expression levels of *cyxA* at 37°C and 41°C were significantly higher compared to 30°C, and the Δ*cyxA* strain exhibited significantly lower viable counts, elevated intracellular reactive oxygen species (ROS) levels, and decreased total antioxidant capacity (T-AOC) relative to WT at 37°C and 41°C. Results from intraperitoneal and intestinal infection models in mice revealed that CyxA promotes pathogenicity by enhancing the invasiveness of *K. pneumoniae* into intra-abdominal tissues and confers a fitness advantage in the inflamed gut. Moreover, we provide preliminary evidence that CyxA exhibits catalase activity and increases the expression of catalase KatE. In summary, our results suggest that cytochrome bd-II oxidase CyxA enhances *K. pneumoniae*’s resistance to oxidative stress caused by exogenous ROS, elevated temperature, and inflammation, either by directly or indirectly metabolizing H_2_O_2_, thereby promoting its growth and pathogenicity.

## Introduction

*Klebsiella pneumoniae* is a leading cause of nosocomial infections (including bacteremia, urinary tract infections, and pneumonia) and community-acquired invasive infections (including endophthalmitis, meningitis, necrotizing fasciitis, osteomyelitis, splenic abscess, and pyogenic liver abscess) [1–4]. The rising incidence of multidrug-resistant *K. pneumoniae* has intensified this public health threat [5], particularly due to the global spread of carbapenem-resistant hypervirulent strains, which often causes untreatable infections [6, 7]. It is widely found in diverse environments, including water, soil, medical devices, as well as the oral cavity and intestinal tract of mammals [2,8].

Oxidative stress is a common environmental challenge for bacteria, arising from an imbalance between ROS and antioxidant defenses [9]. ROS can damage cellular proteins, nucleic acids, and lipids, ultimately leading to cell death [10]. For *K. pneumoniae*, oxidative stress is a significant hurdle in both external environments and infected hosts [11]. During infection, macrophages produce bursts of ROS to eliminate pathogens, while neutrophils, recruited early in the immune response, also generate ROS to combat *K. pneumoniae* [12,13]. Oxidative stress is a key factor in inflammatory bowel disease (IBD), with ROS driving the overproduction of pro-inflammatory cytokines, which in turn leads to ROS accumulation [14]. Our previous study revealed that *K. pneumoniae* exhibits enhanced intestinal colonization and extraintestinal dissemination in the inflamed gut [15]. Additionally, environmental factors such as elevated temperatures and UV irradiation induce rapid ROS accumulation, resulting in oxidative damage to bacteria [16,17]. Despite the importance of these oxidative stresses, there is a notable lack of research on the mechanisms *K. pneumoniae* employs to counteract ROS. Elucidating these resistance mechanisms is crucial for understanding *K. pneumoniae* pathogenicity and informing new therapeutic targets.

Cytochrome bd oxidase is a respiratory quinol: O_2_ oxidoreductase with high oxygen affinity, found in many pathogenic bacteria. Two types of cytochrome bd oxidases have been identified: cytochrome bd-I oxidase CydAB and cytochrome bd-II oxidase CyxAB [18]. In addition to its role in energy generation, cytochrome bd oxidase protects pathogens against various environmental stressors, such as hypoxia, elevated temperatures, and toxic compounds including antibiotics, NO, cyanide, and hydrogen sulfide [19]. Notably, cytochrome bd oxidase enhances bacterial resistance to ROS produced by the host immune system, such as hydrogen peroxide and peroxynitrite [20]. Studies have demonstrated that cytochrome bd oxidase contributes to bacterial virulence through its dual functions of bioenergetics and stress resistance [18]. It has been found to increase the pathogenicity of several bacteria, including *Salmonella typhimurium, Escherichia coli*, *Mycobacterium tuberculosis*, *Listeria monocytogenes* [21–24]. Importantly, cytochrome bd oxidase is exclusive to prokaryotes and absent in eukaryotes, making it a promising target for developing novel antimicrobials. However, despite its significance, the role of cytochrome bd oxidase in *K. pneumoniae* remains largely unexplored.

Herein, we aimed to explore the role of cytochrome bd-II oxidase CyxA in *K. pneumoniae*’s response to oxidative stress and its overall pathogenicity. Our results reveal that CyxA confers an adaptive advantage to *K. pneumoniae* in the abdominal cavity and inflamed gut by resisting oxidative stress, thereby enhancing the pathogenicity. Furthermore, we provide preliminary evidence that CyxA exhibits catalase activity and promotes catalase KatE expression, thereby bolstering oxidative stress resistance in *K. pneumoniae*.

## Materials and methods

### Bacterial strains and culture conditions

Bacterial strains, plasmids, and primers used are listed in Supplementary Tables 1 and 2. All strains were cultured in Luria – Bertani (LB) medium. *K. pneumoniae* strains were standardized as follows: Bacteria were revived from −80°C, cultured overnight at 37°C with shaking at 200 rpm, streaked to isolate single colonies, and grown in fresh LB with 1% inoculum to mid-log phase (optical density at 600 nm (OD_600_) = 1.2). For *in vitro* anaerobic growth analysis, LB liquid medium was aliquoted into screw-cap anaerobic tubes, and oxygen was removed by sparging nitrogen gas through a long needle. The tubes were sealed with rubber stoppers and screw caps, autoclaved, and stored for later use. Standardized *K. pneumoniae* strains were aseptically inoculated into pre-reduced anaerobic LB tubes containing 0, 5, or 15 μM H_2_O_2_, either alone or in combination with 0.4 mM KNO_3_, using a sterile syringe. After 6 h of anaerobic incubation, cultures were serially diluted 10-fold, plated onto LB agar, and incubated overnight at 37°C. Viable bacterial counts were determined by colony enumeration. The study comprised two phases: an initial phase from March 2021 to March 2023, and a supplementary experimental phase conducted in August 2025.

### Construction of traceless deletion mutants and complemented strain

Traceless deletion of *cyxA* from *K. pneumoniae* NTUH-K2044 (WT) was achieved using homologous recombination as described previously [25]. The flanking sequences of *cyxA* were cloned into the temperature-sensitive suicide plasmid pKO3-km (carrying *sacB* and kanamycin resistance genes) to generate pKO3-*cyxAud*. The plasmid pKO3-*cyxAud* was electroporated into WT using a MicroPulser Electroporator (Bio-Rad, Hercules, CA, USA) with the following parameters: 2.5 kV, 25 μF, 200 Ω, 1 mm cuvette. Transformants were selected on LB agar (25 μg/mL kanamycin) at 43°C (non-permissive temperature for plasmid replication). Positive transformants were identified by colony PCR using external primer pairs *cyxA*-A/*cyxA*-D, followed by subculturing at 30°C on sucrose media to eliminate the plasmid via *sacB*-mediated counter-selection. To construct the complemented strain (C-*cyxA*), a 2,432-bp *cyxA* fragment (promoter, open reading frame, and terminator) was cloned into pGEM-T-easy-km (pGEM-*cyxA*) and electroporated into Δ*cyxA*. The flanking sequences of *katE* were cloned into pKO3-km to create pKO3-*katEud*, which was electroporated into WT to generate Δ*katE*. Similarly, the flanking sequences of *katG* were cloned into pKO3-km to create pKO3-*katGud*, which was subsequently electroporated into Δ*katE* to generate the double-knockout strain Δ*katE*/*katG*. The plasmid pGEM-*cyxA* was subsequently transformed into Δ*katE/katG* to obtain the overexpression strain Δ*katE/katG*+*cyxA*. The successful construction of all mutants was confirmed by colony PCR and DNA sequencing.

### Reverse transcription PCR (RT-PCR) and quantitative real-time PCR (qRT-PCR)

Total RNAs were extracted from standardized *K. pneumoniae* strains using the Bacterial RNA Kit (Omega Bio-tek). Genomic DNA was removed, and cDNA was synthesized with the QuantiTect Reverse Transcription Kit (Qiagen). RT-PCR was performed using cDNA templates with 16S rRNA as the internal reference, and gene co-transcription was analyzed via agarose gel electrophoresis. A negative control reaction omitting reverse transcriptase was used to rule out potential genomic DNA contamination during RNA extraction. Relative gene expression levels were quantified by qRT-PCR using SYBR Green Supermix (Bio-Rad), with normalization to the reference gene *recA* and analysis using the comparative ΔΔ*C*_*T*_ method [26]. The reactions were carried out in a 20 μL mixture containing 500 ng of cDNA using the CFX96 Real-Time PCR Detection System (Bio-Rad, Hercules, CA, USA).

### Cytochrome bd oxidase activity assay

Cytochrome bd oxidase activity was measured by *N,N,N,’N’*-tetramethyl-p-phenylenediamine (TMPD, Aladdin) oxidation, a substrate highly specific to bd oxidase [27]. Standardized *K. pneumoniae* strains were cultured in 150 mL of LB medium at 37°C until mid-log phase (OD_600_ = 1.2). The cells were harvested by centrifugation, washed twice with 0.02 M potassium phosphate buffer (pH 7.5, KPB), and resuspended in a 50-mL centrifuge tube. The suspension was sonicated on ice to disrupt cells, followed by centrifugation at 27,000 × g for 30 min at 4°C to collect the supernatant. The supernatant was further ultracentrifuged at 70,000 × g for 2 h, and the resulting membrane pellet was resuspended and homogenized in 0.02 M KPB. Protein concentration in membrane samples was quantified using the BCA Protein Assay Kit (Beyotime) according to the manufacture’s instruction and normalized. Membrane extracts were mixed with TMPD solution (1% TMPD, 0.16 mM ascorbate) and incubated at room temperature. A control reaction was set up by mixing KPB with TMPD solution. The absorbance of reaction mixture was measured at 611 nm (OD_611_), with an increase in OD_611_ reflecting the oxidation of TMPD. Relative cytochrome bd oxidase activity was normalized to the WT strain (100%).

### Oxidative stress assay

Oxidative stress assays were performed as described by Lehman et al. [28], with some modifications. Standardized *K. pneumoniae* strains were cultured in LB medium with 0, 0.1, 0.5, 2 or 4 mM H_2_O_2_ at 37°C for 3 h. Bacterial cultures were serially diluted 10-fold, spotted onto LB plates, and incubated overnight. Colony-forming units (CFU) were then counted to quantify viable bacteria. The survival rate was calculated as the ratio of CFU at different H_2_O_2_ concentrations to the corresponding CFU at 0 mM H_2_O_2_. For NO stress, standardized *K. pneumoniae* strains were grown in LB medium with 0 or 0.5 mM NOC-18 (a NO donor, Aladdin) at 37°C. After 5 h of incubation, cultures were serially diluted 10-fold, spotted onto LB plates, and CFU were calculated. The survival rate was determined by dividing the CFU in 0.5 mM NOC-18 by the corresponding CFU in 0 mM NOC-18. For superoxide anion stress, standardized strains were grown in LB medium with 0 or 0.1 mM methyl viologen (MV, Aladdin) at 37°C for 3 h. The CFU and survival rates were determined as described above. Meanwhile, standardized strains were centrifuged at 12,000 × g for 5 min, washed once with precooled phosphate-buffered saline (PBS), and adjusted to 1 × 10^7^ CFU/mL. The cells were suspended in 100 μL of superoxide dismutase (SOD) sample preparation solution and sonicated on ice to lyse the cells. The resulting lysates were centrifuged at 12,000 × g for 5 min at 4°C to collect the supernatants. Protein concentrations were quantified using the BCA Protein Assay Kit. SOD activity was detected using the Total Superoxide Dismutase Assay Kit with WST-8 (Beyotime) according to the manufacture’s instruction. Detailed of the assay procedures are provided in Supplementary Material 2.

### Catalase activity assay

Standardized *K. pneumoniae* strains were centrifuged at 12,000 × rpm for 10 min, washed twice with precooled PBS, and sonicated on ice to lyse the cells. The resulting lysates were centrifuged at 12,000 × rpm for 5 min at 4°C to collect the supernatants. Protein concentrations were quantified using the BCA Protein Assay Kit. Catalase activity was measured using the Catalase Assay Kit (Beyotime) according to the manufacture’s instruction. Detailed of the assay procedures are provided in Supplementary Material 2.

### Temperature adaptability assay

Standardized *K. pneumoniae* strains were cultured in LB broth and LB plates at 37°C and 30°C, respectively. Growth curves were determined using an automatic growth curve analyzer (Bioscreen, Helsinki, Finland). The maximum biomass, maximum specific growth rate and doubling time were determined using R package grofit (version 1.1.1–1) as previously described [29,30]. The WT, Δ*cyxA* and C-*cyxA* strains were cultured overnight, transferred into fresh LB medium, and grown to the mid-log phase (OD_600_ = 1.2) at 30°C. Cultures were diluted to 10^6^ CFU/mL in LB medium and incubated under static conditions at 20°C, 30°C or 37°C for 3 h, or at 41°C for 1.5 h. Following incubation, cultures were serially diluted 10-fold and plated on LB agar to calculate CFU. For RNA analysis, the WT strain was cultured in LB broth and treated at 30°C, 37°C or 41°C. Total RNAs were extracted after 2 and 4 h of treatment. The relative expression levels of the *cyxA* gene were quantified by qRT-PCR.

Relative levels of ROS were measured using the ROS Assay Kit (Beyotime). Bacterial cultures were adjusted to 10^7^ CFU/mL in PBS and incubated with 10 μM 2′,7′-dichlorodihydrofluorescein diacetate (DCFH-DA) at 37°C for 20 min in the dark. After washing twice, bacteria were resuspended in LB broth and subjected to temperature treatments as above. Fluorescence was detected using a microplate reader (BioTek, Vermont, USA) with excitation at 488 nm and emission at 525 nm. Detailed of the assay procedures are provided in Supplementary Material 2. ROS levels were normalized to those at 30°C. Total antioxidant capacity was determined using the T-AOC Assay Kit (Solarbio). Briefly, standardized *K. pneumoniae* strains were collected, suspended in 1 mL of precooled extraction solution, and sonicated on ice to lyse the cells. The resulting lysates were centrifuged at 10,000 × rpm for 10 min at 4°C to collect the supernatants. Protein concentrations were quantified using the BCA Protein Assay Kit. A working solution was prepared before use by mixing the three reagents in a proportion of 7:1:1 (v/v). The reaction was performed at room temperature for 10 min in a 1,020-μL reaction mixture containing 900 μL working solution, 30 μL sample, and 90 μL ddH_2_O. The absorbance of reaction mixture was measured at 593 nm. The T-AOC value was normalized to the total protein level.

### Mouse intraperitoneal infection experiments

Six-week-old female specific-pathogen-free (SPF) BALB/c mice were purchased from HUNAN SJA Laboratory Animal Co., Ltd (Hunan, China). All mice were randomly divided into several groups. *K. pneumoniae* strains were standardized and adjusted to 10^4^ CFU/mL in PBS. Mice were intraperitoneally injected with 100 μL of bacterial suspension containing 10^3^ CFU of WT (*n* = 21), Δ*cyxA* (*n* = 21), C-*cyxA* (*n* = 6), or with PBS as a control (*n* = 3). Survival rates were monitored over 14 days.

In a separate experiment, three groups of BALB/c mice were intraperitoneally injected with 10^3^ CFU of WT (*n* = 8), Δ*cyxA* (*n* = 8) or C-*cyxA* (*n* = 6). At 24 h post-infection, mice were deeply anesthetized via intraperitoneal injection of pentobarbital sodium (50 mg/kg) for terminal blood collection. Euthanasia was immediately performed by cervical dislocation under deep anesthesia, in accordance with the American Veterinary Medical Association’s guidelines. Blood samples were smeared on LB agar for bacterial counts. Peritoneal lavage fluids (PLF) were collected by injecting 3 mL of LB broth into the abdominal cavity, mixed for 1 min, and analyzed by gram staining and microscopy (OLYMPUS, Tokyo, Japan). Ten-fold serial dilutions of 20 μL PLF were plated for bacterial enumeration. The liver, spleen and lung tissues were aseptically removed, weighed, and homogenized in 2 mL of PBS using a tissue homogenizer (Biospec, California, USA). The homogenates were serially diluted (1:10), and plated on LB agar to determine bacterial loads. All mice were included in the analysis, except those that died from non-experimental causes. The corresponding authors were aware of the group allocations at each stage of the experiment.

### Mouse intestinal infection experiments

Eight-week-old female SPF C57BL/6 mice were purchased from the Experimental Animal Center of Hubei University of Medicine. To induce an IBD model, mice were administered 3% (w/v) dextran sulfate sodium (DSS; relative molecular mass 36,000– 50,000; MP Biomedicals) in sterilized distilled water for 10 days, while control mice (Mock) received sterilized water. Body weight and fecal observations were monitored daily. DSS was replaced with sterilized water one day before the end of the experiment. On day 7, following a 4 h fast, mice were intragastrically inoculated with 100 μL of WT, Δ*cyxA* (10^9^ CFU, *n* = 5), or PBS (*n* = 3). At 24 and 48 h post-infection, feces were collected, weighed, suspended in PBS, serially diluted, and plated for bacterial counts. On day 3 post-infection, mice were sacrificed, and blood was collected for serum isolation by centrifuging at 1,000 × g for 15 min at 4°C. Serum levels of calprotectin (CALP) and intestinal fatty acid binding protein (iFABP) were determined using ELISA kits (CUSABIO) according to the manufacture’s instruction. Detailed of the assay procedures are provided in Supplementary Material 2. The large intestine and small intestine were collected, weighed, homogenized in PBS, serially diluted, and plated to determine bacterial loads. Colon lengths were measured to assess inflammation-induced shortening. Colon tissues were fixed in paraformaldehyde, embedded in paraffin, sectioned using a microtome, and stained with hematoxylin and eosin for histopathological examination.

### Statistical analyses

Statistical comparisons between two groups were performed using an unpaired two-tailed Student’s *t* test. Comparisons among three or more groups were conducted using one-way ANOVA. Survival data were analyzed using the Kaplan–Meier method and the log-rank (Mantel-Cox) test. A *p-*value < 0.05 was considered statistically significant.

### Ethics statement

All animal experiments were complied strictly with the ARRIVE guidelines (https://arriveguidelines.org/), and were approved by the Animal Care and Use Committee of Hubei University of Medicine (Reference Number: HBMU 2020–103 and 2025–133).

## Results

### Effect of oxidative stress on the growth of *K. pneumoniae* and cytochrome bd oxidase gene expression

**Figure 1.**
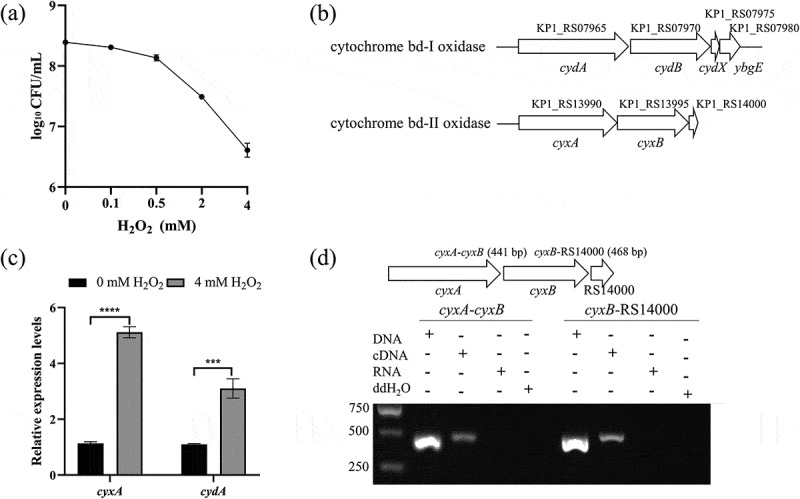
Effect of H_2_O_2_ stress on *K. pneumoniae* survival and the expression of cytochrome bd oxidase. (a) Viable bacterial counts of the wt strain were measured after growth in liquid lb medium with 0, 0.1, 0.5, 2 or 4 mM H_2_O_2_ at 37 °c for 3 hours with 200 rpm shaking. (b) Gene clusters encoding cytochrome bd-I and bd-II oxidase in the NTUH-K2044 genome. (c) qRT-PCR analysis of *cyxA* and *cydA* gene expression in the wt strain cultured in lb broth with 0 mM or 4 mM H_2_O_2_ for 3 hours. (d) Co-transcription analysis of *cyxA*, *cyxB* and KP1_RS14000 genes, determined by RT-PCR of fragments between adjacent genes. Data are presented as mean ± standard deviation (sd) from three independent experiments. ***, *p* < 0.001; ****, *p* < 0.0001 (unpaired two-tailed Student’s *t* test).

To explore the resistance of *K. pneumoniae* to oxidative stress, we assessed the survival rates of the WT strain exposed to different concentrations of H_2_O_2_. The viable bacterial counts in LB medium decreased from 2.47 × 10^8^ CFU/mL (0 mM H_2_O_2_) to 2.03 × 10^8^, 1.37 × 10^8^, 3.1 × 10^7^ and 4.17 × 10^6^ CFU/mL at H_2_O_2_ concentrations of 0.1, 0.5, 2 and 4 mM, respectively (Figure 1(a)). The genome of *K. pneumoniae* NTUH-K2044 encodes two types of cytochrome bd oxidases: cytochrome bd-I oxidase (encoded by *cydAB*) and cytochrome bd-II oxidase (encoded by *cyxAB*) (Figure 1(b)). qRT-PCR analysis revealed that *cyxA* expression significantly increased 4.1-fold, while *cydA* expression increased 2.1-fold after treatment with 4 mM H_2_O_2_ (Figure 1(c)), implying that *cyxA* gene plays a more prominent role in oxidative stress resistance. The fragments between *cyxA*, *cyxB* and the adjacent gene KP1_RS14000 were amplified successfully by RT-PCR using primers targeting regions between adjacent genes (Figure 1(d)), suggesting that these genes are co-transcribed and likely belong to the same operon. These results indicate that *K. pneumoniae* has a certain ability to resist oxidative stress, with cytochrome bd oxidases, especially bd-II oxidase, may play an important role in this resistance.

### Loss of cyxA weakens the tolerance of *K. pneumoniae* to exogenous oxidative stress

**Figure 2.**
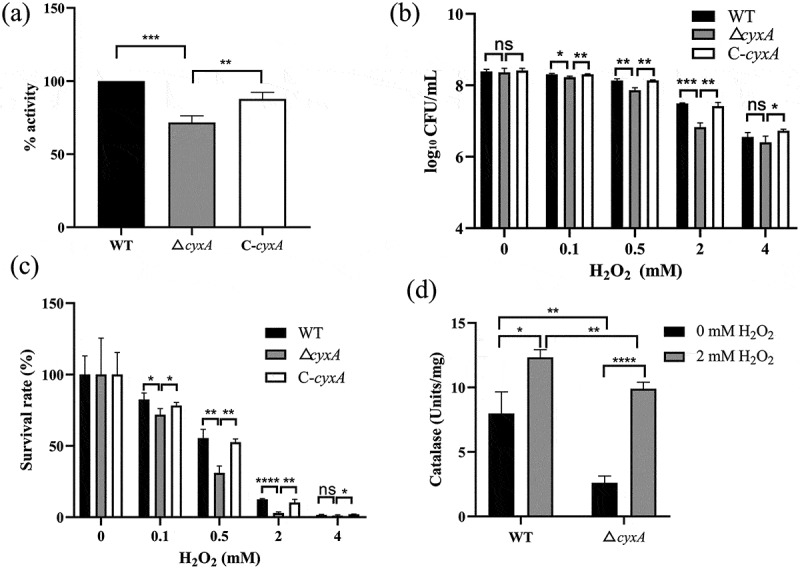
Effect of the *cyxA* gene on cytochrome bd oxidase activity and sensitivity of *K. pneumoniae* to H_2_O_2_. (a) The relative activity of cytochrome bd oxidase in the wt, Δ*cyxA*, and C-*cyxA* strains was calculated by measuring the oxidation levels of TMPD using membrane extracts from log-phase cells incubated at room temperature. (b) Viable bacterial counts of the WT, Δ*cyxA* and C-*cyxA* strains grown in lb liquid medium containing different concentrations of H_2_O_2_ at 37 °c for 2 hours with 200 rpm shaking. (c) Survival rates of the WT, Δ*cyxA* and C-*cyxA* strains under different concentrations of H_2_O_2_. (d) Catalase activity in the WT and Δ*cyxA* strains grown in Lb liquid medium with 0 or 2 mM H_2_O_2_ at 37 °c for 2 hours, measured using the Catalase Assay Kit (Beyotime). Data are presented as mean ± sd from three independent experiments. *, *p* < 0.05; **, *p* < 0.01; ***, *p* < 0.001; ****, *p* < 0.0001; ns, not significant (a–c: one-way anova; d: unpaired two-tailed Student’s *t* test).

To explore the function of *cyxA* gene in *K. pneumoniae*, a traceless deletion mutant (Δ*cyxA*) and a complemented strain (C-*cyxA*) were constructed. The successful construction of the Δ*cyxA* and C-*cyxA* strains was confirmed by RT-PCR, and deletion of *cyxA* did not affect the expression of the adjacent gene *cyxB* (Supplementary Figure S1), ruling out any polarity effects caused by gene knockout. Membrane extracts from log-phase cells of the WT, Δ*cyxA* and C-*cyxA* strains were used to assess cytochrome bd oxidase activity by measuring TMPD oxidation. The relative activity of cytochrome bd oxidase in Δ*cyxA* was significantly lower than that in WT and C-*cyxA* (Figure 2(a)), suggesting that CyxA is required for cytochrome bd-II oxidase activity.

The expression level of *cyxA* gene in *K. pneumoniae* increased remarkably under oxidative stress conditions (Figure 1(c)), implying that *cyxA* may play a role in oxidative stress resistance. To further explore this, the growth and survival of different strains were compared after exposure to H_2_O_2_, NO, and superoxide anions. In LB medium containing 0.1, 0.5, or 2 mM H_2_O_2_, both the viable bacterial count and survival rate of Δ*cyxA* were significantly lower than those of WT, whereas C-*cyxA* exhibited similar levels to WT (Figure 2(b,c)). These results confirm that CyxA contributes to the resistance of *K. pneumoniae* to H_2_O_2_. Furthermore, the catalase activity of WT and Δ*cyxA* strains with or without H_2_O_2_ was measured. H_2_O_2_ treatment increased catalase activity in both WT and Δ*cyxA* strains (Figure 2(d)). However, Δ*cyxA* showed significantly lower catalase activity than WT under both treated and untreated conditions (Figure 2(d)), which likely explains its reduced resistance to H_2_O_2_.

**Figure 3.**
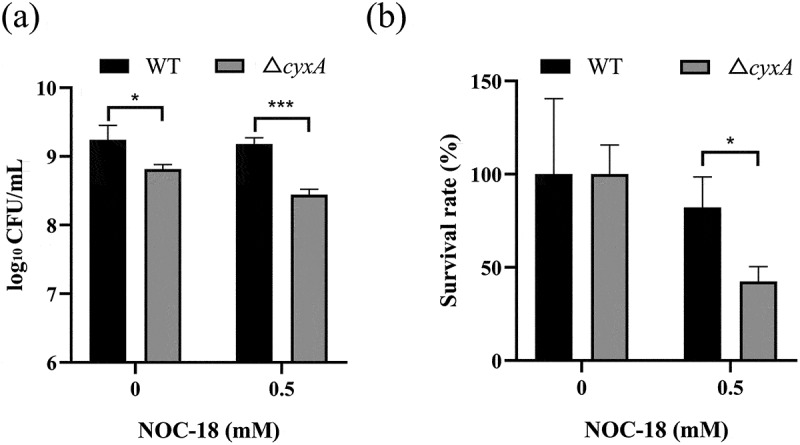
Sensitivity of the WT and Δ*cyxA* strains to no. (a) Viable bacterial counts of the WT and Δ*cyxA* strains grown in lb medium with 0 or 0.5 mM NOC-18 (a no donor) for 5 hours. (b) Survival rates of the wt and Δ*cyxA* strains under NOC-18 treatment. Data are presented as mean ± sd from three independent experiments. *, *p* < 0.05; ***, *p* < 0.001 (unpaired two-tailed Student’s *t* test).

To assess resistance to NO, NOC-18 was added. Although the viable bacterial count of Δ*cyxA* was lower than that of WT under both treated and untreated conditions (Figure 3(a)), its survival rate under NOC-18 treatment was significantly reduced compared to WT (42.4% versus 82.1%, Figure 3(b)). These results confirm that the loss of *cyxA* reduces the resistance of *K. pneumoniae* to NO. To assess resistance to superoxide anions, MV was added. Although the viable bacterial count of Δ*cyxA* was lower than that of WT under both treated and untreated conditions (Supplementary Figure S2a), the survival rate under MV treatment and SOD activity showed no significant difference between WT and Δ*cyxA* (Supplementary Figure S2b-c), suggesting that *cyxA* does not affect the resistance of *K. pneumoniae* to superoxide anions. Taken together, these results indicate that CyxA is critical for the resistance of *K. pneumoniae* to exogenous H_2_O_2_ and NO but not to superoxide anions.

### CyxA enhances the high-temperature adaptability of K. pneumoniae by modulating antioxidant capacity

**Figure 4.**
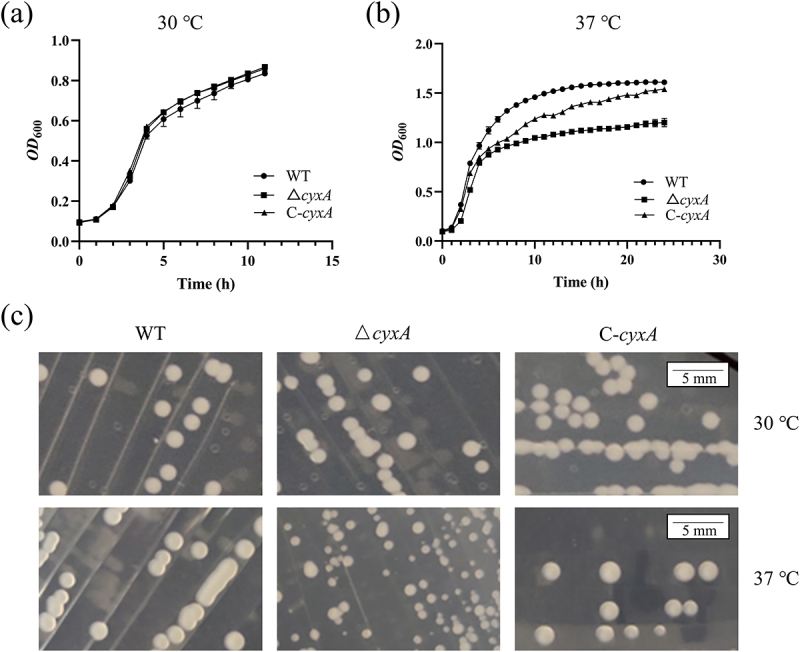
Effect of the *cyxA* gene on the growth and colony morphology of *K. pneumoniae*. (a) Growth curves of the wt, Δ*cyxA* and C-*cyxA* strains in lb medium at 30°C. (b) Growth curves of the wt, Δ*cyxA* and C-*cyxA* strains in lb medium at 37°C. (c) Colony morphology of the wt, Δ*cyxA* and C-*cyxA* strains on lb agar plates at 30°C and 37°C. Data are presented as mean ± SD from three independent experiments.

*E. coli* deficient in cytochrome bd oxidase exhibits temperature-sensitive growth defects [31]. To assess the role of CyxA in the temperature adaptability of *K. pneumoniae*, we compared the aerobic growth of the WT, Δ*cyxA* and C-*cyxA* strains at 37°C and 30°C. At 30°C, all strains exhibited comparable growth profiles (Figure 4(a)), with no significant differences in maximum biomass, maximum specific growth rate or doubling time between Δ*cyxA* and WT (Supplementary Figure S3a-c). In contrast, at 37°C, Δ*cyxA* showed impaired growth during both the log and stationary phases (Figure 4(b)), with a significantly reduced maximum biomass, maximum specific growth rate and a prolonged doubling time compared to WT (Supplementary Figure S3d-f). The growth of C-*cyxA* was partially restored at 37°C (Figure 4(b)). On LB plates, colonies appeared normal for all strains at 30°C, but Δ*cyxA* formed microcolonies at 37°C (Figure 4(c)). We speculate that the growth defect of Δ*cyxA* at 37°C may be related to its reduced temperature adaptability.

**Figure 5.**
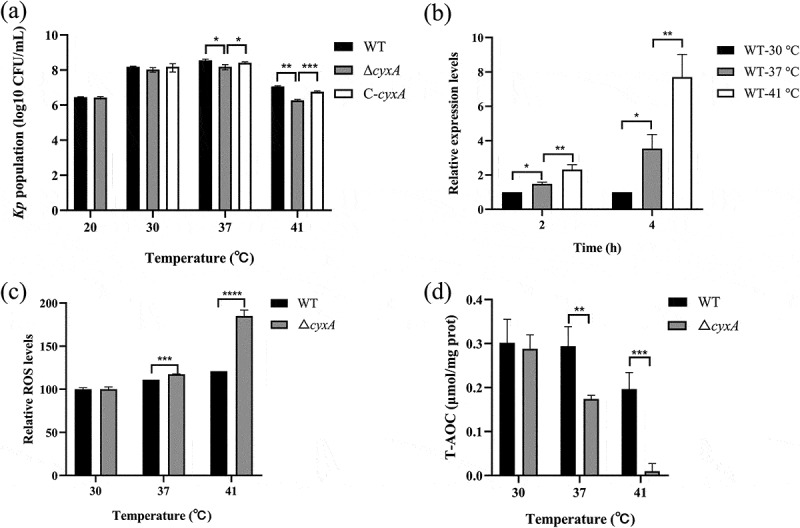
Effect of the *cyxA* gene on the high-temperature adaptability of *K. pneumoniae*. (a) Viable bacterial counts of the wt, Δ*cyxA* and C-*cyxA* strains cultured in lb medium at various temperatures. (b) Relative expression levels of the *cyxA* gene measured by qRT-PCR in the wt strain after 2 and 4 hours of incubation at 30°C, 37 °c and 41°C. (c) Relative levels of ros in wt and Δ*cyxA* strains cultured at 30°C, 37°C and 41°C, as measured by the ros assay Kit (Beyotime). (d) T-AOC in wt and Δ*cyxA* strains cultured at 30°C, 37°C and 41°C, as measured by the T-AOC assay Kit (Solarbio). Data are presented as mean ± sd from three independent experiments. *, *p* < 0.05; **, *p* < 0.01; ***, *p* < 0.001; ****, *p* < 0.0001 (unpaired two-tailed Student’s *t* test).

To further explore the relationship between the *cyxA* gene and temperature adaptability, viable bacterial counts were measured for the WT, Δ*cyxA* and C-*cyxA* strains at different temperatures. At 37°C and 41°C, both WT and C-*cyxA* exhibited significantly higher viable counts compared to Δ*cyxA*. Notably, the reduction in viability between WT and Δ*cyxA* was more pronounced at 41°C (6.2-fold; *p* < 0.01) than at 37°C (2.3-fold; *p* < 0. 05). No significant differences were observed at 20°C and 30°C (Figure 5(a)). qRT-PCR analysis showed that the expression levels of *cyxA* in WT were significantly increased at 37°C (2 h: 1.5-fold, 4 h: 3.5-fold; *p* < 0.05) and 41°C (2 h: 2.3-fold, 4 h: 7.7-fold; *p* < 0.01) compared to 30°C, and the expression levels increased with rising temperatures (Figure 5(b)). These results demonstrate the role of *cyxA* in promoting the adaptability of *K. pneumoniae* at higher temperatures.

Elevated temperatures reduce dissolved oxygen levels and increase ROS production [17,32,33]. To investigate the mechanism, intracellular ROS levels and antioxidant capacity were measured in the WT and Δ*cyxA* strains at different temperatures. Intracellular ROS levels increased with temperature in both strains. However, Δ*cyxA* exhibited significantly higher ROS levels than WT at 37°C (1.1-fold; *p* < 0.001) and 41°C (1.5-fold; *p* < 0.0001). Notably, the difference became more pronounced at higher temperatures (Figure 5(c)). Correspondingly, Δ*cyxA* showed a marked reduction in T-AOC compared to WT at 37°C (*p* < 0.01) and 41°C (*p* < 0.001). Furthermore, in our experiments, T-AOC in Δ*cyxA* was almost undetectable at 41°C (Figure 5(d)). Taken together, the results indicate that CyxA enhances the high-temperature adaptability of *K. pneumoniae* by modulating its antioxidant capacity.

### CyxA provides adaptive advantages for *K. pneumoniae* in intraperitoneal and inflammatory intestinal infections

**Figure 6.**
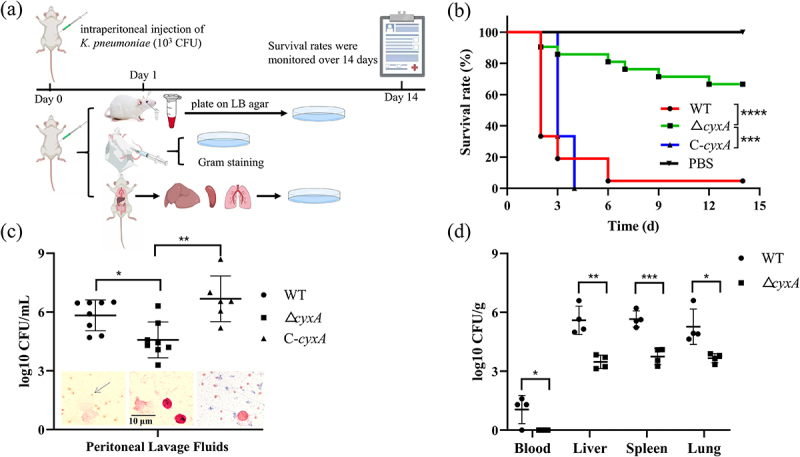
Effect of the *cyxA* gene on mice following intraperitoneal infection with *K. pneumoniae*. Groups of BABL/c mice were injected intraperitoneally with 10^3^ cfu of wt (*n* = 21), Δ*cyxA* (*n* = 21), C-*cyxA* (*n* = 6) or pbs (*n* = 3). (a) Schematic diagram illustrating the experimental timeline for mouse intraperitoneal infection. (b) Mouse survival was monitored daily for 14 days, and survival curves were plotted. (c) Bacterial loads in plf from mice infected with wt, Δ*cyxA* or C-*cyxA*. At 24 hours post-infection with 10^3^ cfu of wt (*n* = 8), Δ*cyxA* (*n* = 8) or C-*cyxA* (*n* = 6), plf samples were collected for bacterial counting and gram staining. (d) Bacterial loads in the blood, liver, spleen and lungs of mice were determined 24 hours post-infection with wt or Δ*cyxA*. Data are presented as mean ± sd. Results shown in panels b and c represent data from three and two independent experiments, respectively. *, *p* < 0.05; **, *p* < 0.01; ***, *p* < 0.001; ****, *p* < 0.0001 (b: log-rank (Mantel-Cox) test; c–d: unpaired two-tailed Student’s *t* test).

To investigate the effect of *cyxA* gene on the pathogenicity of *K. pneumoniae*, BALB/c mice were intraperitoneally infected with PBS, 10^3^ CFU of WT, Δ*cyxA* or C-*cyxA* (Figure 6(a)). The Δ*cyxA* mutant exhibited attenuated virulence, as evidenced by a significantly higher survival rate in infected mice compared to those infected with WT (*p* < 0.0001) or C-*cyxA* (*p* < 0.001). On day 3, survival rates were 19% and 33% in the WT and C-*cyxA* groups, respectively, compared with 86% in the Δ*cyxA* group. All mice infected with C-*cyxA* succumbed within 4 days. By day 14, survival in the WT group decreased to 5%, while survival in the Δ*cyxA* group remained at 67%. No significant difference in survival was observed between the WT and C-*cyxA* groups (*p* > 0.05) (Figure 6(b)). To further investigate whether CyxA affects the ability of *K. pneumoniae* to defend against peritoneal immune responses, the survival abilities of WT, Δ*cyxA* and C-*cyxA* strains in the mouse peritoneal cavity were examined. At 24 h post-infection, the PLF were collected for Gram staining and bacterial counting. The results revealed abundant *K. pneumoniae* in the PLF of WT- and C-*cyxA*-infected mice, whereas no bacteria were detectable in Δ*cyxA*-infected mice. Meanwhile, WT- and C-*cyxA*-infected mice exhibited significantly more bacterial counts in PLF than Δ*cyxA*-infected mice, with no significant difference between WT- and C-*cyxA* groups (Figure 6(c)). Furthermore, the blood, livers, spleens and lungs of mice were collected for bacterial counting to explore whether CyxA affects the invasive ability of *K. pneumoniae*. WT-infected mice had detectable bacteria in blood, whereas Δ*cyxA*-infected mice exhibited no detectable bacteria in blood. Besides, the bacterial loads in the livers, spleens and lungs of WT-infected mice were all significantly higher than those in Δ*cyxA*-infected mice (liver: 130-fold, spleen: 81-fold, lung: 40-fold; Figure 6(d)). These findings suggest that loss of *cyxA* significantly decreases the defense against peritoneal immune responses and the invasive ability of *K. pneumoniae*, thus reducing its pathogenicity during intraperitoneal infection.

To determine whether CyxA functions in intestinal infection, C57BL/6 mice were infected intragastrically with 10^9^ CFU of WT, or Δ*cyxA* (Supplementary Figure S4a). No significant differences in bacterial loads were observed in the feces, large intestine, or small intestine between the WT and Δ*cyxA* groups (Supplementary Figure S4b-c). Levels of CALP and iFABP, both markers of intestinal damage [14,34], were comparable between the infected groups (Mock+WT and Mock+Δ*cyxA*) and the control group (Mock+PBS; Supplementary Figure S4d). Additionally, HE staining showed no obvious intestinal lesions in the infected groups (Supplementary Figure S4e). In conclusion, these results indicate that CyxA does not have a significant impact in the normal intestinal anaerobic environment.

**Figure 7.**
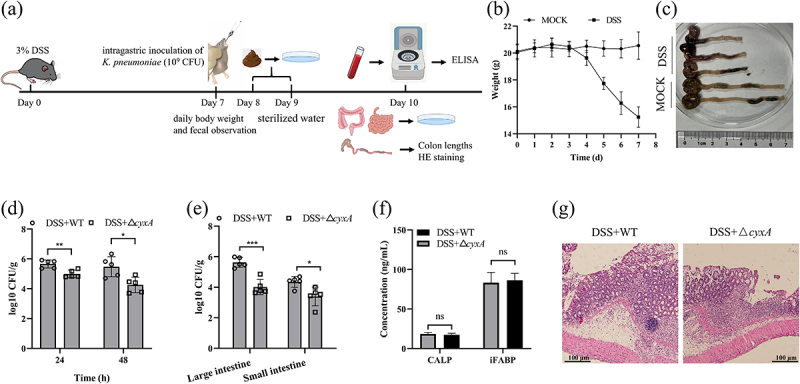
Effect of the *cyxA* gene on mice with ibd intragastrically infected with *K. pneumoniae*. Groups of C57BL/6 mice were given 3% DSS (DSS group) or water (mock group) and subsequently inoculated intragastrically with 10^9^ cfu of wt (*n* = 5), Δ*cyxA* (*n* = 5) on day 7. Mice were sacrificed on day 3 post-infection. (a) Schematic diagram illustrating the experimental timeline for mouse inflammatory intestinal infection. (b) Daily body weight changes of mice during 7 days of DSS or water treatment. (c) Colon lengths of DSS-treated and mock-treated mice were measured. (d) Fecal bacterial counts in DSS-treated mice were determined at 24 and 48 hours post-infection with wt or Δ*cyxA*. (e) Bacterial loads in the large intestine and small intestine of DSS-treated mice were assessed on day 3 post-infection with wt or Δ*cyxA*. (f) Serum concentrations of calp and iFABP were measured by elisa (CUSABIO). (g) Colon tissues were collected, sectioned, and stained with hematoxylin and eosin (H&E). Data are presented as mean ± SD. *, *p* < 0.05; **, *p* < 0.01; ***, *p* < 0.001; ns, not significant (unpaired two-tailed Student’s *t* test).

To explore the role of CyxA in *K. pneumoniae* infections of the inflammatory intestine, an IBD model was established using DSS (Figure 7(a)). The mice exhibited significant weight loss and shortened colons, confirming successful IBD induction (Figure 7(b,c)). In DSS-treated mice, fecal bacterial counts for the Δ*cyxA*-infected group were 5-fold and 17-fold lower at 24 and 48 h, respectively, compared to the WT-infected group (Figure 7(d)). Bacterial loads in the large intestine and small intestine of Δ*cyxA*-infected mice were 41- and 9-fold lower, respectively, than those in WT-infected mice (Figure 7(e)). Serum CALP and iFABP levels, along with histopathological analyses, showed no significant differences between Δ*cyxA*- and WT-infected groups in DSS-treated mice (Figure 7(f,g)). These results indicate that CyxA provides an adaptive advantage for *K. pneumoniae* in inflammatory intestinal infections.

**Figure 8.**
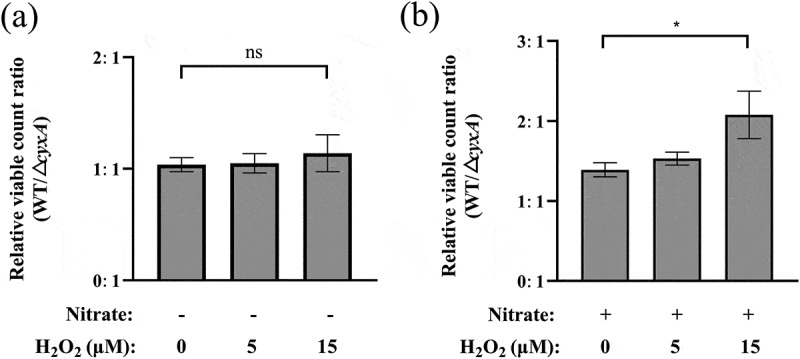
Effect of the *cyxA* gene on the anaerobic growth of *K. pneumoniae* in low concentrations of H_2_O_2_. (a) Relative viable bacterial ratio of the wt and Δ*cyxA* strains after anaerobic cultivation in lb liquid medium with 0, 5, and 15 μM H_2_O_2_ for 6 hours. The ratio was calculated as (WT CFU/mL) divided by (Δ*cyxA* CFU/mL). (b) Relative viable bacterial ratio of the WT and Δ*cyxA* strains after anaerobic cultivation in lb liquid medium with 0, 5, and 15 μM H_2_O_2_ supplemented with 0.4 mM KNO_3_ for 6 hours. Data are presented as mean ± SD from three independent experiments. *, *p* < 0.05; ns, not significant (unpaired two-tailed Student’s *t* test).

The coexistence of nitrate and H_2_O_2_ in inflamed intestines strongly induces bd-II oxidase activity in *E. coli* [35]. To mimic the intestinal inflammatory environment, WT and Δ*cyxA* strains were cultured anaerobically in LB medium containing low concentrations of H_2_O_2_, with or without KNO_3_. In the presence of H_2_O_2_ alone, the growth of WT and Δ*cyxA* was similar (Figure 8(a)). However, when both H_2_O_2_ and KNO_3_ were present, the WT/Δ*cyxA* growth ratio (defined as [WT CFU/mL]/[Δ*cyxA* CFU/mL]) increased dose-dependently with H_2_O_2_ concentration: 1.39 (0 μM H_2_O_2_), 1.53 (5 μM), and 2.08 (15 μM) (Figure 8(b)), indicating a marked growth advantage of WT over Δ*cyxA*. These findings suggest that CyxA confers a growth advantage to *K. pneumoniae* in an inflammatory gut-mimicking environment containing both H_2_O_2_ and nitrate.

### CyxA exhibits catalase activity and increases the expression of the catalase KatE

**Figure 9.**
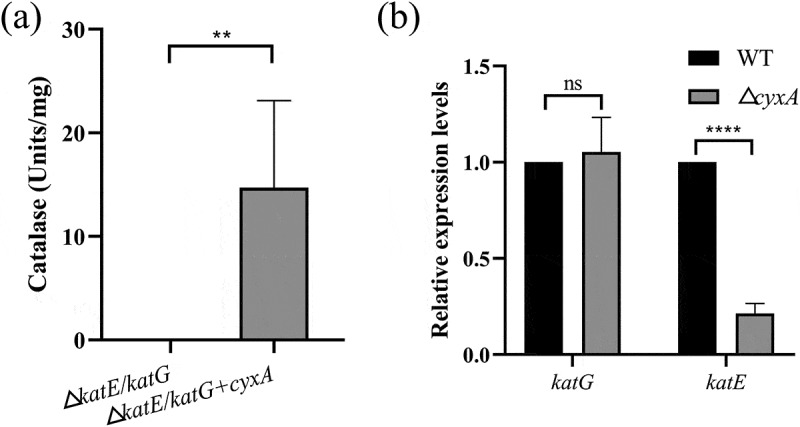
Effect of the *cyxA* gene on the catalase activity of *K. pneumoniae*. (a) Catalase activity in the Δ*katE*/*katG* and Δ*katE*/*katG+cyxA* strains, measured by the Catalase Assay Kit (Beyotime). (b) Relative expression levels of catalase genes *katG* and *katE* in the wt and Δ*cyxA* strains, as determined by qRT-PCR. Data are presented as mean ± SD from three independent experiments. **, *p* < 0.01; ****, *p* < 0.0001; ns, not significant (unpaired two-tailed Student’s *t* test).

SOD and catalase are critical antioxidants in bacteria that eliminate ROS under oxidative stress [19,35]. Our previous findings revealed that loss of *cyxA* reduced catalase activity without affecting SOD activity, suggesting that diminished catalase activity may contribute to the reduced resistance to oxidative stress and pathogenicity after deletion of *cyxA*. To further explore this, a double-knockout strain (Δ*katE/katG*) was constructed by deleting the catalase genes *katE* and *katG*, and the overexpression strain Δ*katE/katG*+*cyxA* was generated by introducing *cyxA* into this strain. Catalase activity in the Δ*katE*/*katG* strain was negligible, whereas overexpression of *cyxA* led to a sharp increase of catalase activity in the Δ*katE/katG*+*cyxA* strain (Figure 9(a)). Moreover, the expression level of *katE* was significantly lower in Δ*cyxA* compared to WT (Figure 9(b)). These results preliminarily demonstrate that CyxA possesses intrinsic catalase activity and enhances the expression of catalase KatE.

## Discussion

Cytochrome bd oxidase, found exclusively in bacteria and archaea, confers resistance to various environmental stresses and contributes to the virulence of several pathogens, making it a promising target for next-generation antibacterial drugs [18–20,36]. Some bacteria possess two types of cytochrome bd oxidases: bd-I oxidase (CydAB) and bd-II oxidase (CyxAB), with the physiological role of bd-II oxidase remaining incompletely understood [19,35]. The *K. pneumoniae* K2044 genome encodes both types of bd oxidases: *cydA* (KP1_RS7965), *cydB* (KP1_RS7970) and *cyxA* (KP1_RS13990), *cyxB* (KP1_RS13995). However, the specific function of cytochrome bd oxidase in *K. pneumoniae* remains unclear. Therefore, we constructed a traceless *cyxA* deletion mutant and a complemented strain to investigate the role of cytochrome bd-II oxidase (CyxA) in *K. pneumoniae*. Our results demonstrate that CyxA enhances *K. pneumoniae* resistance to exogenous ROS, elevated temperature, and inflammatory conditions, thereby protecting the bacteria from oxidative stress and promoting both growth and pathogenicity. Additionally, we provide preliminary evidence that CyxA possesses catalase activity and boosts the expression of catalase KatE, potentially contributing to the decomposition of harmful substances in oxidative stress.

ROS-induced oxidative stress is a common challenge for bacteria during host invasion. It has been proved that bd oxidase contributes to degrading ROS produced by the host immune system [18,19]. Consistent with this, our study found that deletion of *cyxA* reduced *K. pneumoniae* resistance to exogenous H_2_O_2_ and NO. In *E. coli*, bd-I oxidase knockout mutants exhibit high sensitivity to H_2_O_2_ exposure, with bd-I oxidase expression increasing in response to exogenous H_2_O_2_ [37]. Similarly, uropathogenic *E. coli* strains lacking bd oxidase display significantly increased sensitivity to both H_2_O_2_ and NO [38]. This protective effect of bd oxidase has also been observed in *Brucella*, *Porphyromonas gingivalis*, *M. tuberculosis*, and *Mycobacteria smegmatis* [19,39]. It has been reported that *E. coli* bd-I oxidase possesses catalase-like and peroxidase-like activities, while purified bd-II oxidase shows high catalase activity, enabling direct metabolism of H_2_O_2_ [19,40]. We proved that *K. pneumoniae* CyxA can metabolize H_2_O_2_ both directly and indirectly to defend against H_2_O_2_-induced oxidative damage, potentially explaining its role in promoting bacterial growth under oxidative stress. However, the precise mechanisms by which CyxA metabolizes H_2_O_2_ need further research.

Cytochrome bd oxidase, a respiratory terminal oxidase in prokaryotic electron transport chains, has varying effects on the growth of different pathogens [18]. In *M. tuberculosis* and *L. monocytogenes*, only one type of bd oxidase is present. While the deletion of *cydAB* does not affect the growth of *M. tuberculosis*, it leads to an aerobic growth defect in *L. monocytogenes* [24,41]. In contrast, *S. typhimurium* and *E. coli* possess two types of bd oxidases. For *S. typhimurium*, the *cydA* gene provides a fitness advantage under 8% oxygen, whereas the *cyxA* gene is specifically required for growth in 0.8% oxygen [42]. In *E. coli*, bd-I oxidase contributes to both microaerobic respiration and aerobic growth, while bd-II oxidase functions under extremely low oxygen levels without affecting aerobic growth [35,43]. Our study revealed that deletion of *cyxA* affected the aerobic growth of *K. pneumoniae* at 37°C and formed microcolonies, a result similar to observations in *E. coli cydAB* mutant [44].

Besides, bacteria encounter temperature fluctuations during host invasion and tissue migration. The temperatures of bloodstream, lung, liver, and intestine range from 37°C to 38°C. Inflammatory responses can further raise body temperature to 39°C−41°C [45,46]. Elevated temperatures often lead to excessive ROS production, causing oxidative damage [17]. In *E. coli*, cytochrome bd oxidase mitigates high-temperature- induced ROS, promoting bacterial proliferation; while its absence results in temperature-sensitive growth defects [31]. Similarly, our findings demonstrate that bd-II oxidase CyxA enhances the high-temperature adaptability of *K. pneumoniae* by modulating its antioxidant capacity. Deletion of *cyxA* impairs the ROS-scavenging capacity of *K. pneumoniae* at 37°C, resulting in a slight increase in intracellular ROS levels and a corresponding reduction in bacterial viability. These results indicate that CyxA is required for optimal growth of *K. pneumoniae* at 37°C. While 37°C is the optimal growth temperature, it imposes a greater oxidative burden than 30°C, necessitating CyxA-mediated antioxidant defenses. Furthermore, at 41°C – a temperature that induces oxidative stress – deletion of *cyxA* exacerbates intracellular ROS accumulation, accompanied by a more pronounced reduction in viability.

Using different animal models, we investigated the effects of CyxA on *K. pneumoniae* pathogenicity. Our results revealed that CyxA increases pathogenicity by enhancing the invasiveness of *K. pneumoniae* in intra-abdominal tissues (including the liver, spleen, and lung) and promotes bacterial adaptation in the inflamed gut, but not in the normal intestine. Reports have shown that cytochrome bd oxidase is essential for *in vivo* survival of *Vibrio vulnificus* and *Brucella abortus* during intraperitoneal infection [47,48]. Cytochrome bd-I oxidase CydA enhances *Salmonella* virulence in murine models of intraperitoneal infection through its dual role in bioenergetics and antinitrosative defense [21]. Several studies have reported elevated ROS levels in the enteric cavity during gut inflammation, with the subsequent degradation of ROS into oxygen enabling *E. coli* bd oxidases (CydAB and CyxAB) to mediate aerobic respiration and confer a fitness advantage in the inflamed gut [35,49,50]. Cytochrome bd-II oxidase CyxA supports *S. typhimurium* expansion in the antibiotic-treated gut [42]. Similarly, CydAB-mediated aerobic respiration supports the expansion of *Citrobacter rodentium* in the mouse colon [51]. In our study, deletion of *cyxA* led to the most significant fitness defect in *K. pneumoniae* during intraperitoneal infection, compared to intestinal infections. Although the Δ*cyxA* strain exhibited both reduced oxidative stress resistance and a growth defect at 37°C *in vitro*, the tissue-specific differences in bacterial *in vivo* fitness may be attributed to impaired oxidative stress resistance. Oxidative stress is generally greater in the peritoneal infection model compared to the intestinal infection model, even though both sites are maintained at 37°C [52–54]. The increased oxidative stress in the peritoneal model arises from intense immune-mediated ROS production, limited local antioxidant capacity, and systemic inflammatory cascades, whereas the intestinal tract balances immunity and redox homeostasis through mucosal barriers, low-oxygen metabolism, and microbial symbiosis [12,54,55].

By simulating the intestinal inflammatory environment *in vitro*, we found that CyxA may confer a fitness advantage for *K. pneumoniae* during anaerobic growth through respiration using H_2_O_2_ as a substrate. This aligns with observations in *E. coli*, where an inflammatory intestinal environment significantly increases bd-II oxidase activity, with catalase-mediated ROS degradation producing oxygen that serves as the terminal electron acceptor for bd-II oxidase, thereby promoting *E. coli* growth during gut inflammation [35]. Collectively, our *in vitro* and *in vivo* findings suggest that cytochrome bd-II oxidase CyxA enhances *K. pneumoniae* pathogenicity mainly by resisting and even utilizing ROS.

Lee et al. demonstrated that the cytochrome bd oxidase inhibitor ND-011992 synergizes with the cytochrome bcc:aa_3_ oxidase inhibitor Q203 to effectively kill both replicating and antibiotic-tolerant, non-replicating mycobacteria, showing greater efficacy compared to monotherapy [56]. These results, combined with our findings, suggest that developing inhibitors targeting cytochrome bd oxidases represents a promising therapeutic strategy for *K. pneumoniae* infections. This approach holds particular significance in addressing the urgent clinical need for effective treatments against carbapenem-resistant, hypervirulent *K. pneumoniae*. Our study elucidates the role and mechanism of cytochrome bd-II oxidase CyxA in *K. pneumoniae* infection, providing a foundation for future inhibitor development.

## Supplementary Material

supplementary figure S1.tif

supplementary figure S4.tif

supplementary figure S3.tif

supplementary figure S2.tif

Clean Copy of Supplementary Material - QVIR-2025-0271.R1.docx

## Data Availability

The data that support the findings of this study are openly available in ScienceDB at https://doi.org/10.57760/sciencedb.23498, reference number [57].
