# Supplementary figures and images for: Cytochrome bd-II oxidase CyxA promotes the pathogenicity of Klebsiella pneumoniae by resisting oxidative stress
Source: Virulence. 2025 Nov 14;16(1):2590244. doi: 10.1080/21505594.2025.2590244 (PMC12629338; doi:10.1080/21505594.2025.2590244)

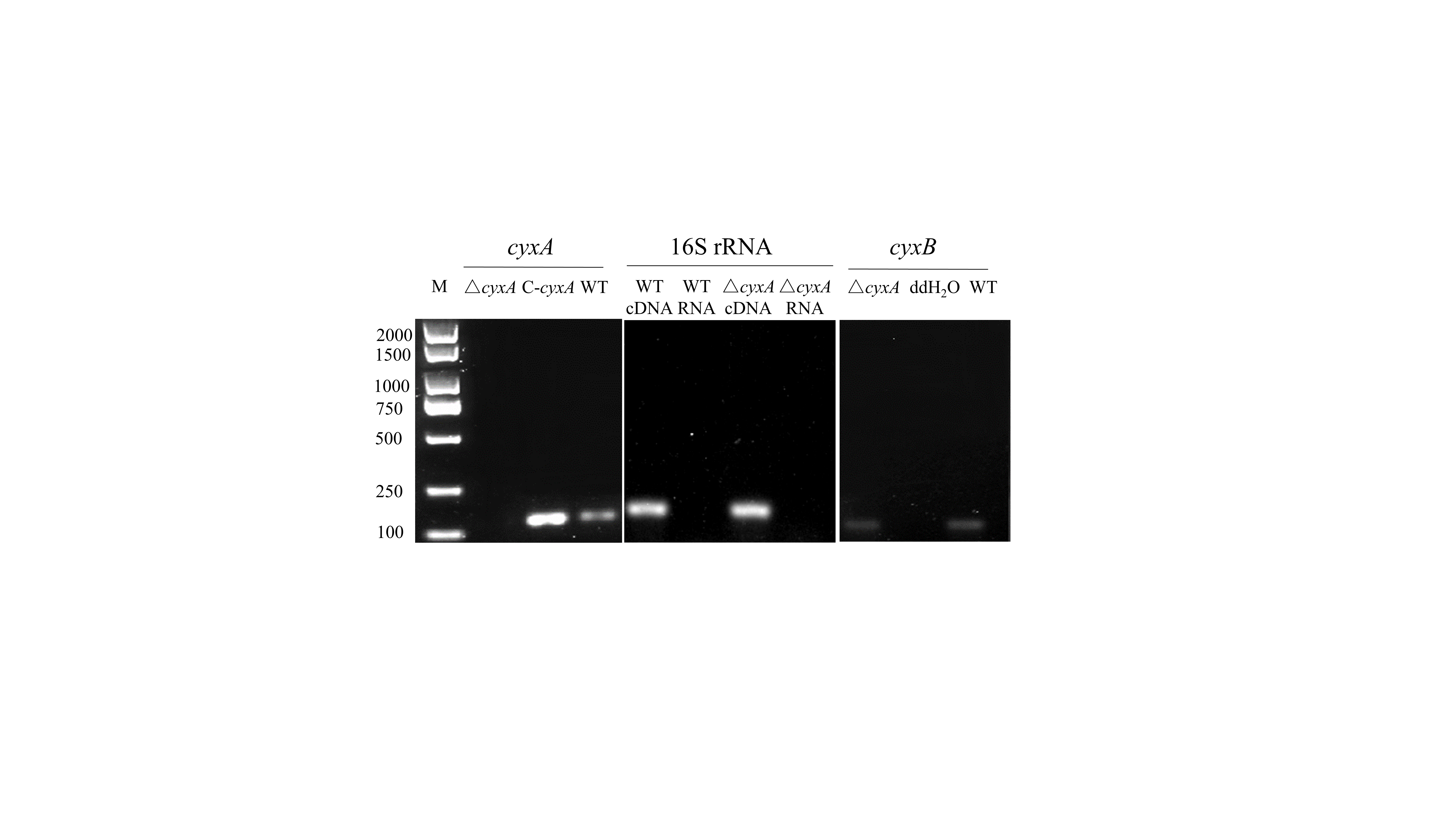

Supplement: supplementary figure S1.tif [file KVIR_A_2590244_SM8215.tif]

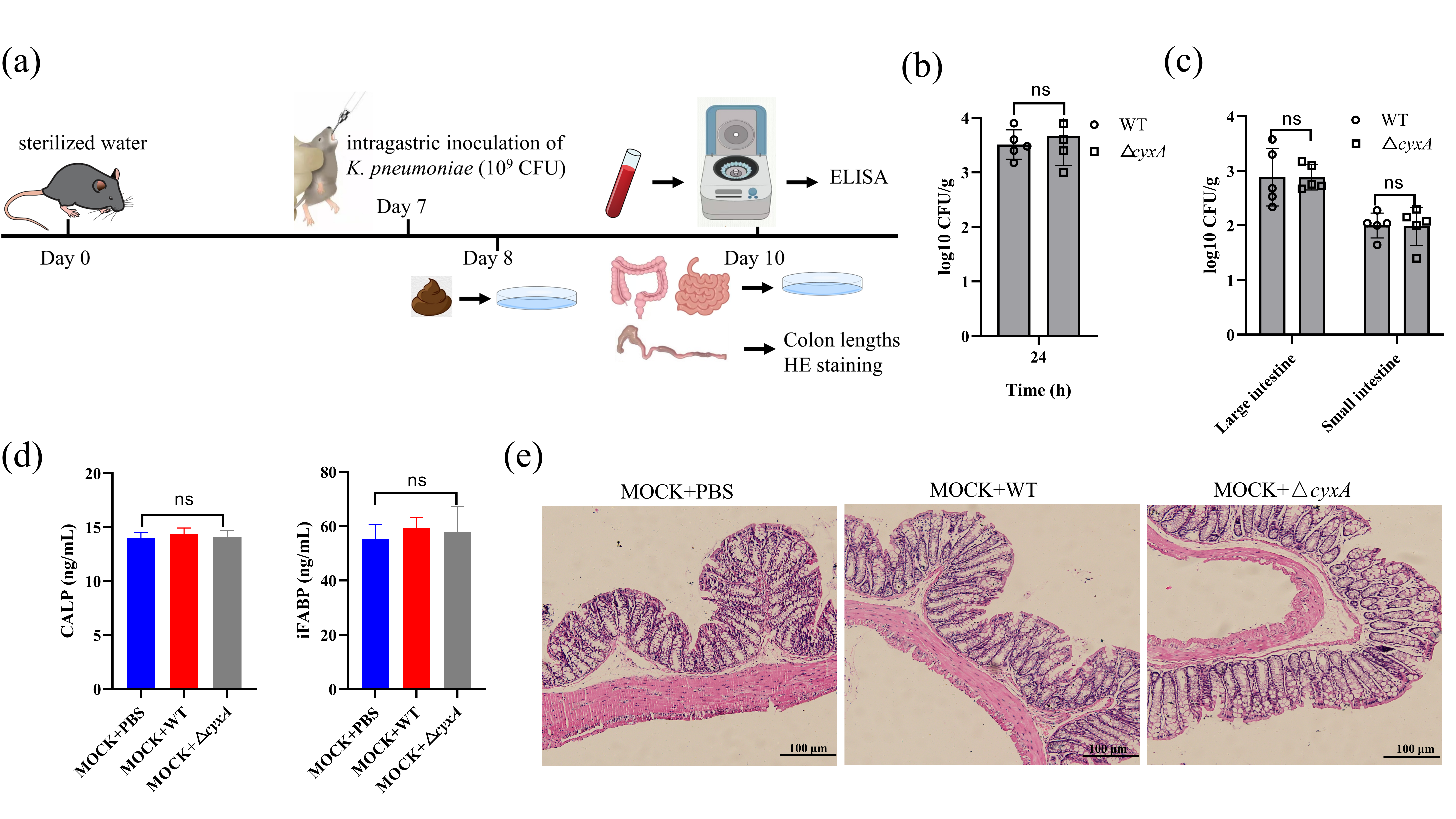

Supplement: supplementary figure S4.tif [file KVIR_A_2590244_SM8214.tif]

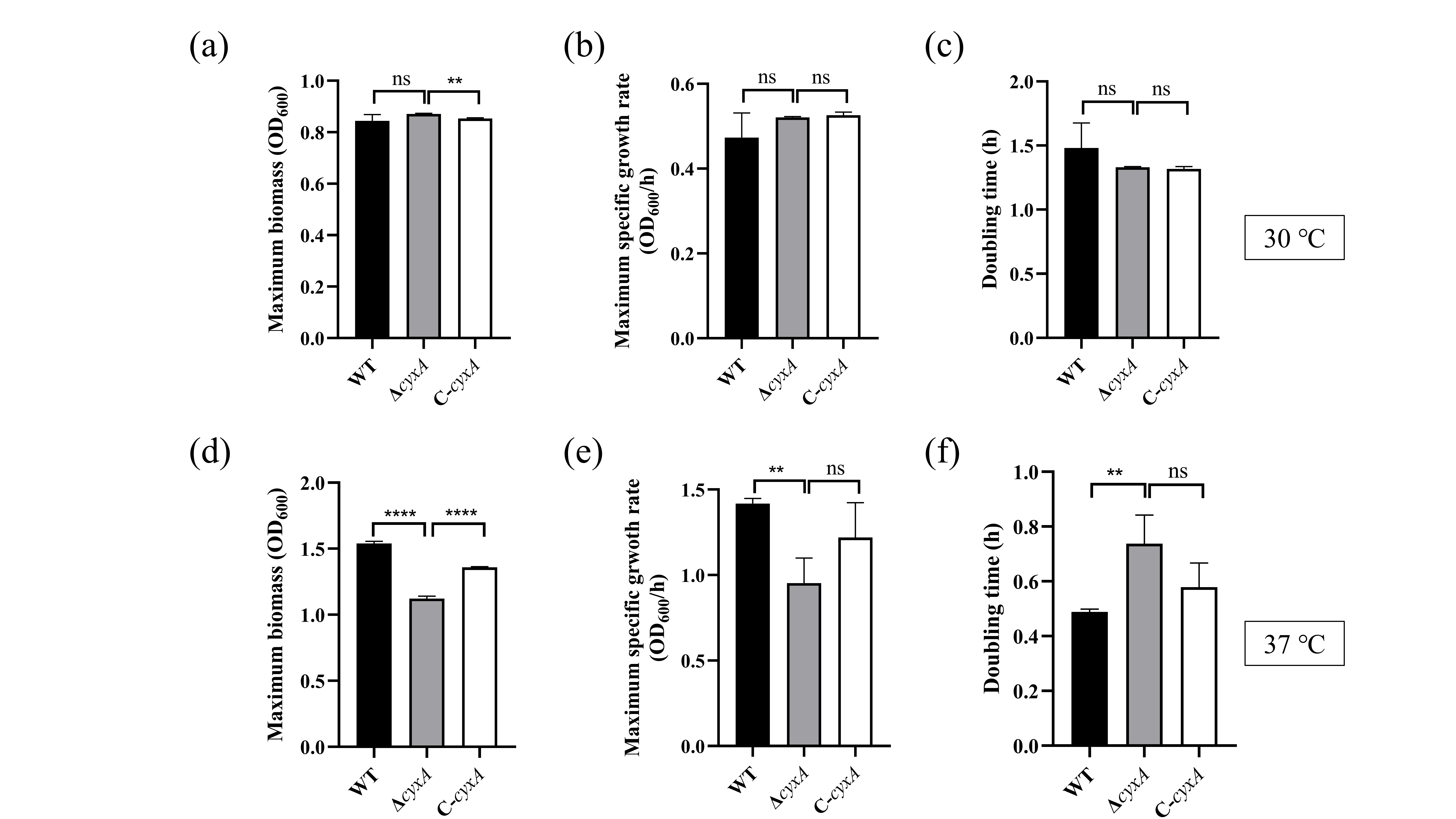

Supplement: supplementary figure S3.tif [file KVIR_A_2590244_SM8213.tif]

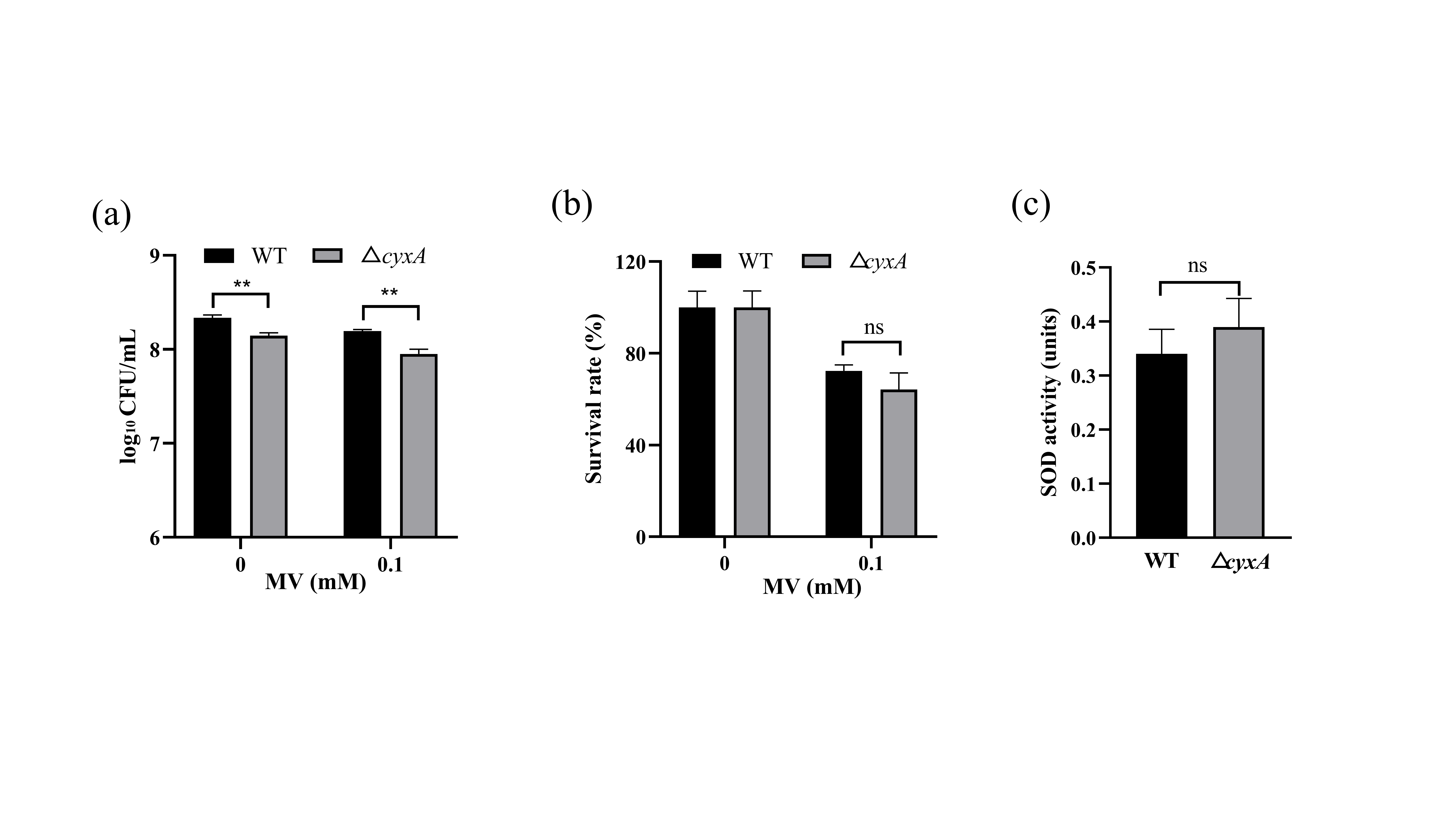

Supplement: supplementary figure S2.tif [file KVIR_A_2590244_SM8212.tif]
