# Supplementary material for: Cytochrome bd-II oxidase CyxA promotes the pathogenicity of Klebsiella pneumoniae by resisting oxidative stress
Source: Virulence. 2025 Nov 14;16(1):2590244. doi: 10.1080/21505594.2025.2590244 (PMC12629338; doi:10.1080/21505594.2025.2590244)
Supplement: Clean Copy of Supplementary Material - QVIR-2025-0271.R1.docx [file KVIR_A_2590244_SM8211.docx]

**Supplementary Figure Legends**

**
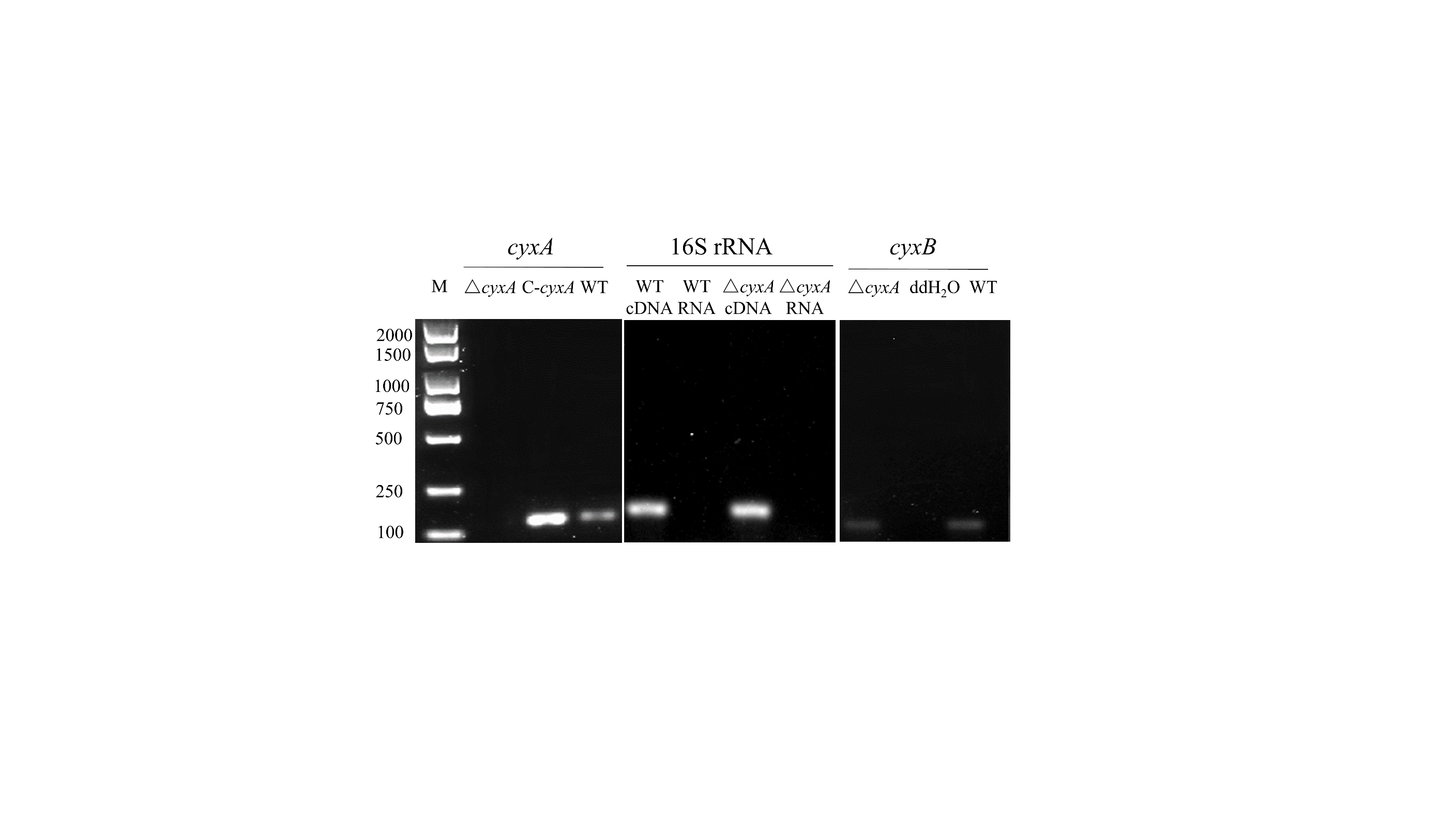
**

**Supplementary Figure 1.** **Identification of the *cyxA* gene deletion strain and complemented strain.** RT-PCR was performed to confirm the identification of the Δ*cyxA* and C-*cyxA* strains, as well as to assess the impact of *cyxA* knockout on the adjacent gene.

**
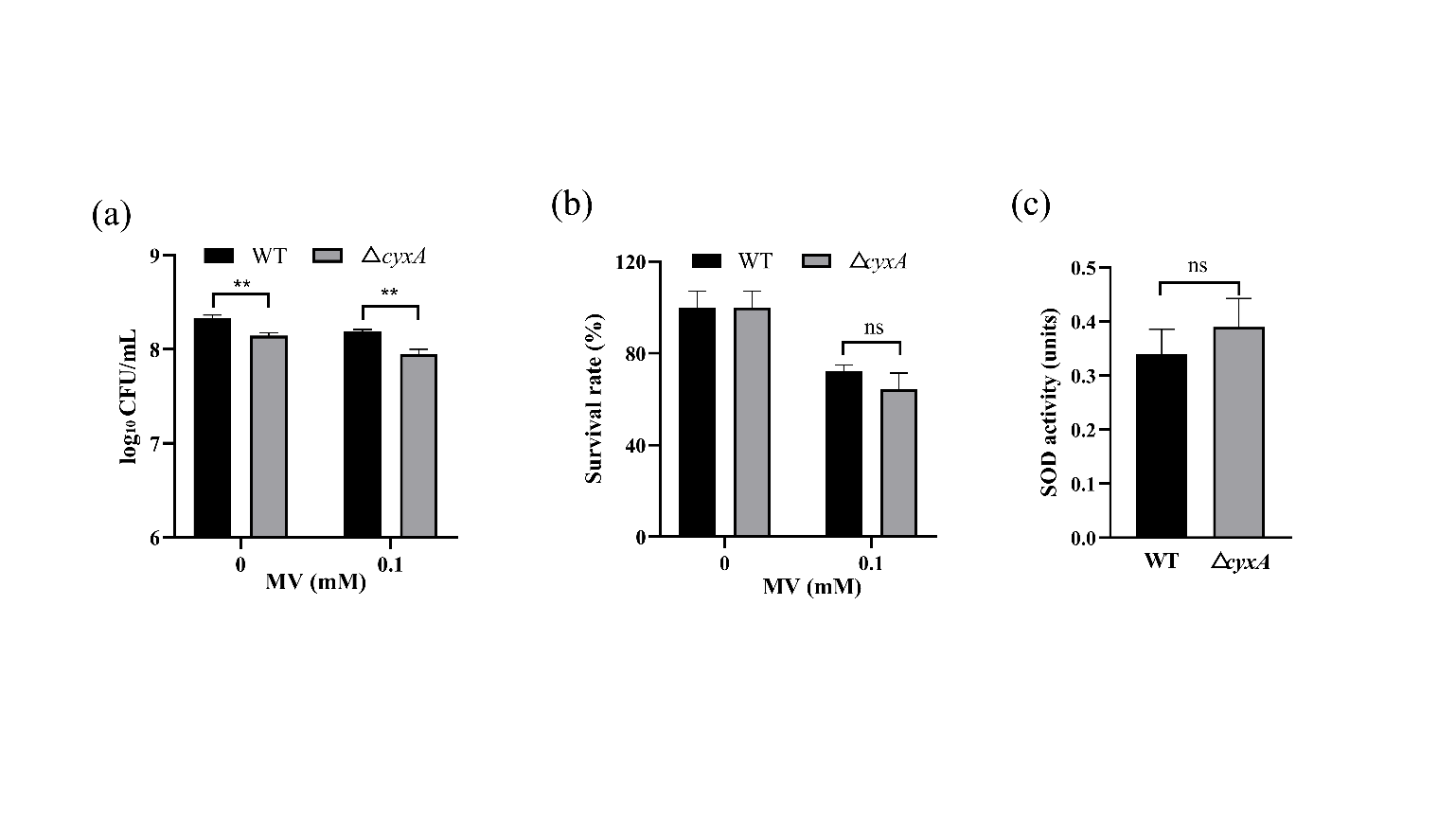
**

**Supplementary Figure 2.** **Sensitivity of the WT and Δ*cyxA* strains to superoxide anion.** (a) Viable bacterial counts of strain WT and Δ*cyxA* grown in LB medium with 0 or 0.1 mM methyl viologen (MV, a superoxide anion generator) for 3 hours. (b) Survival rates of strain WT and Δ*cyxA* under 0 or 0.1 mM MV. (c) Superoxide dismutase (SOD) activity from log-phase cells of strain WT and Δ*cyxA* was measured by the Total SOD Assay Kit with WST-8 (Beyotime). Data are presented as mean ± SD from three independent experiments. **, *P* < 0.01; ns, not significant (unpaired two-tailed Student’s *t* test).


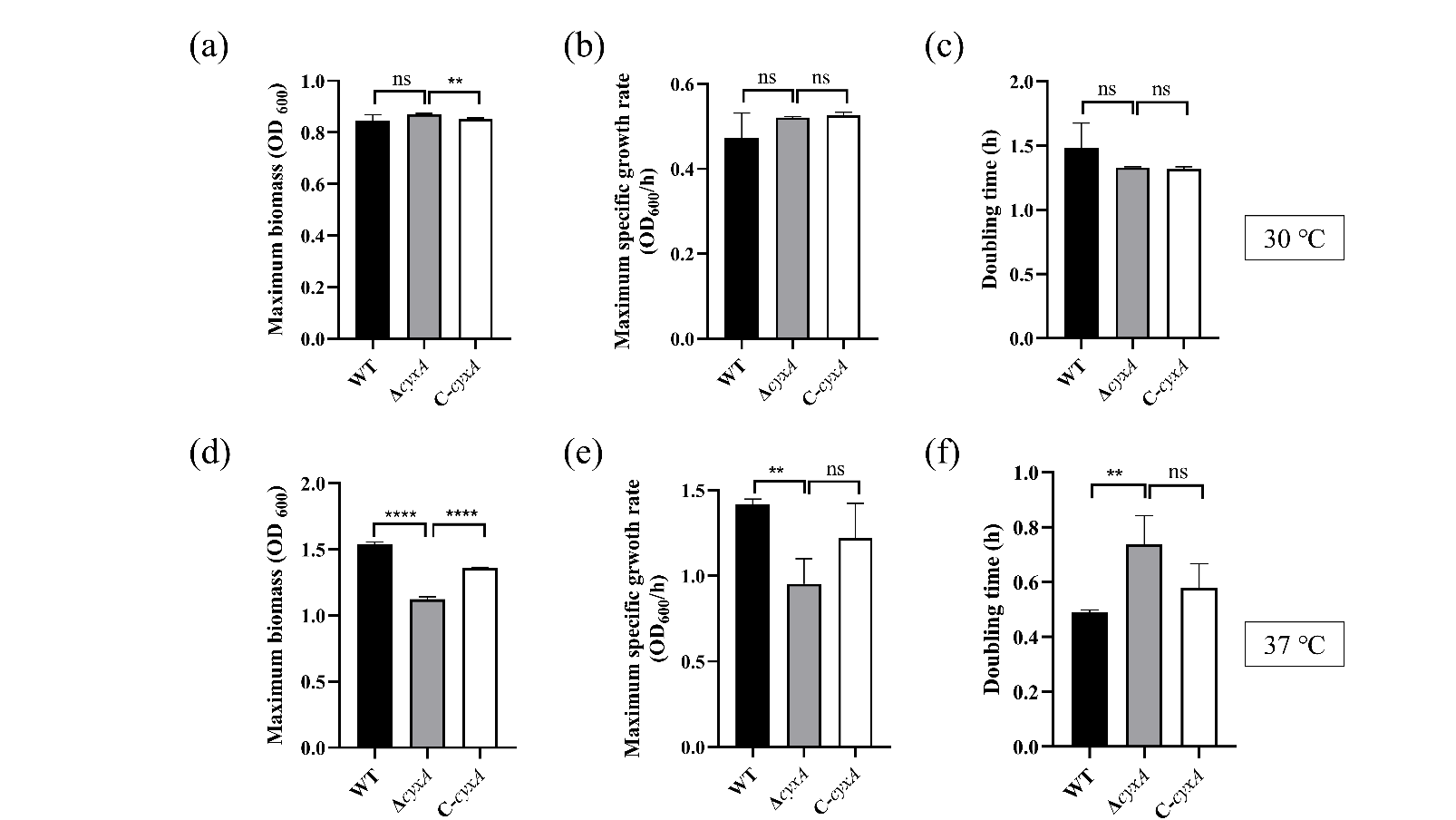


**Supplementary Figure 3.** **Effect of the *cyxA* gene on the growth of *K. pneumoniae*.** Maximum biomasses (a), maximum specific growth rates (b), and doubling times (c) of the WT, Δ*cyxA* and C-*cyxA* strains in LB medium at 30 °C. Maximum biomasses (d), maximum specific growth rates (e), and doubling times (f) of the WT, Δ*cyxA* and C-*cyxA* strains in LB medium at 37 °C. Data are presented as mean ± SD. **, *P* < 0.01; ****, *P* < 0.0001; ns, not significant (unpaired two-tailed Student’s *t* test).


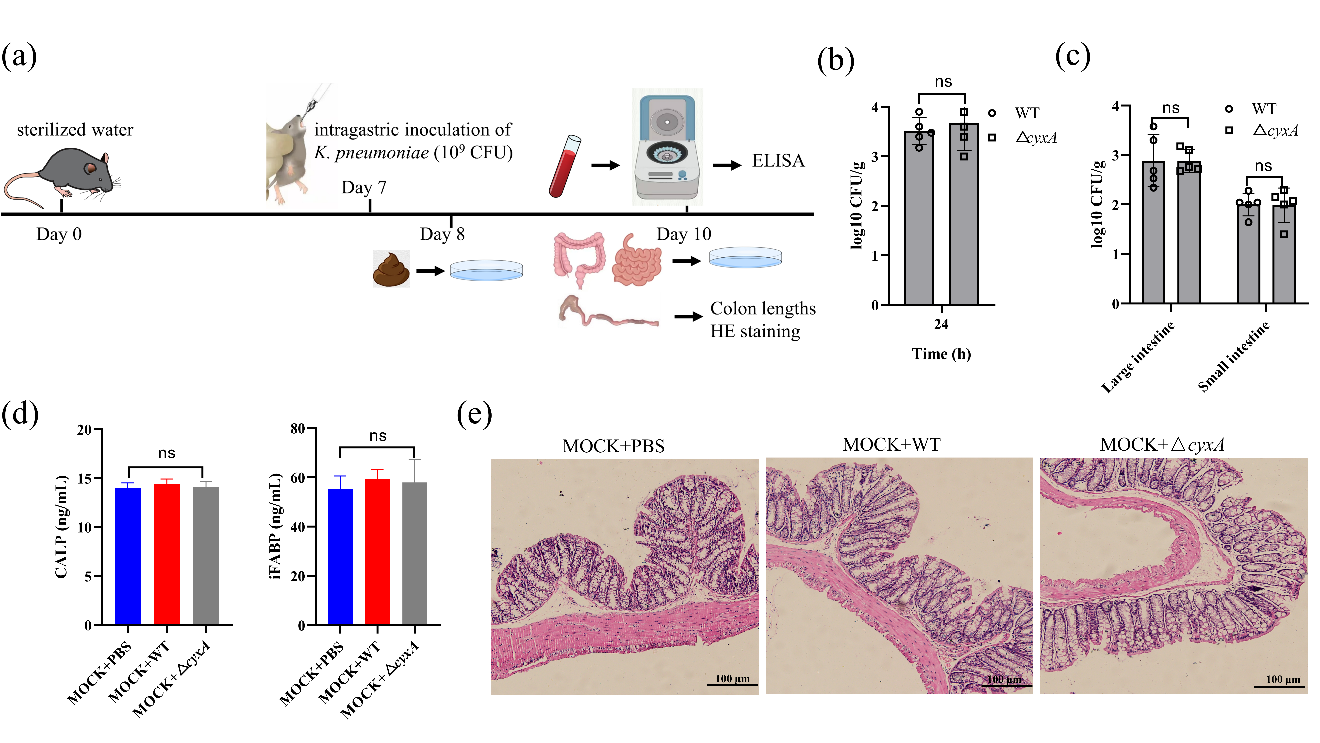


**Supplementary Figure 4.** **Effect of the *cyxA* gene on normal mice intragastrically infected with *K. pneumoniae*.** Groups of C57BL/6 mice were inoculated intragastrically with 10^9^ CFU of WT (*n* = 5), Δ*cyxA* (*n* = 5) or PBS (*n* = 3). Mice were sacrificed on day 3. (a) Schematic diagram illustrating the experimental timeline for mouse intestinal infection. (b) Fecal bacterial counts in normal mice were determined at 24 hours post-infection with WT or Δ*cyxA* strain. (c) Bacterial loads in the large intestine and small intestine of mice were determined on day 3 post-infection with WT or Δ*cyxA*. (d) Serum concentrations of calprotectin (CALP) and intestinal fatty acid binding protein (iFABP) were determined by ELISA (CUSABIO). (e) Colon tissues of mice were collected, sectioned, and stained with hematoxylin and eosin. ns, not significant (unpaired two-tailed Student’s *t* test).

**References**

1. Wang M, Tian Y, Xu L, et al. High osmotic stress increases Ompk36 expression through the regulation of KbvR to decrease the antimicrobial resistance of *Klebsiella pneumoniae.* Microbiol Spectr. 2022;10(3):e0050722. <https://doi.org/10.1128/spectrum.00507-22>
2. Xie J, Wang H, Ma R, et al. The molybdate transport protein ModA regulates nitrate reductase activity to increase the intestinal colonization and extraintestinal dissemination of *Klebsiella pneumoniae* in the inflamed gut*.* Virulence. 2025;16(1):2474185. <http://doi.org/10.1080/21505594.2025.2474185>

**Supplementary Table 1 Bacterial strains and plasmids used in this work**

| Strains or plasmids | Descripton | Reference |
| --- | --- | --- |
| Strains | | |
| *E. coli* DH5α | F- φ80d/lacZΔM15 Δ(*lacZYA*-*argF*) U169 *deoR* *recA*1 *hsdR*17(rk- mk-) *phoA* *supE*44 λ- *thi*-1 *gyrA*96 *relA*1 | [1] |
| *K. pnemoniae* | | |
| NTUH-K2044 (WT) | A hypervirulent capsular serotype K1 strain with hypermucoviscosity phenotype, wild-type strain, Amp^R^ | [2] |
| Δ*cyxA* | Traceless deletion of *cyxA* from WT, Amp^R^ | Constructed in the present study |
| C-*cyxA* | Complemented *cyxA* mutant, Amp^R^, Kan^R^ | Constructed in the present study |
| Δ*katE*/*katG* | Traceless deletion of both *katE* and *katG* from WT, Amp^R^ | Constructed in the present study |
| Δ*katE*/*katG*+*cyxA* | Double-knockout strain Δ*katE*/*katG* containing pGEM-*cyxA*, Amp^R^, Kan^R^ | Constructed in the present study |
| Plasmids | | |
| pKO3-Km | Suicide plasmid, Kan^R^, *sacB* (sucrose-lethal gene), pSC101 temperature-sensitive origin | [2] |
| pKO3-*cyxAud* | Knockout plasmid for the ORF region of *cyxA* | Constructed in the present study |
| pKO3-*katEud* | Knockout plasmid for the ORF region of *katE* | Constructed in the present study |
| pKO3-*katGud* | Knockout plasmid for the ORF region of *katG* | Constructed in the present study |
| pGEM-T-easy-km | pGEM-T-easy with an insertion of Km cassette from pUC4K into the *Nde*I site for trans-complementation, Kan^R^ | [2] |
| pGEM-*cyxA* | pGEM-T-easy-derived vector containing the promoter, ORF and terminator region of *cyxA* | Constructed in the present study |

**Supplementary Table 2 Primers used in this work**

| Primer name | Sequences (5’→3’) | Function |
| --- | --- | --- |
| *cyxA*-A | GTATGCGGCCGCTCAGACATTGGCATTCCCT | Amplification of the *cyxA* left flanking sequence |
| *cyxA*-B | AGATAACCTTCAGTGGCGGGCAGGACTCCACAACAAAAT |  |
| *cyxA*-C | ATTTTGTTGTGGAGTCCTGCccgccactgaaggttatCt | Amplification of the *cyxA* right flanking sequence |
| *cyxA*-D | GTATGCGGCCGCAGCCAGTCAAACATGCCG |  |
| C-*cyxA*-F | AGGGCCCAAATATCACCACCCAGAATAG | Amplification of the promoter, ORF and terminator region of *cyxA* |
| C-*cyxA*-R | ATGCGGCCGCGGTCAGCCAGTCAAACATGC |  |
| 16S rRNA_RT_F | ATGACCAGCCACACTGGAAC | Amplification of 16S rRNA gene |
| 16S rRNA_RT_R | CTTCCTCCCCGCTGAAAGTA |  |
| *cydA*-RT-F | TCATCCTGGCGATTAGCTCC | Amplification of *cydA* gene |
| *cydA*-RT-R | TCGTAACCGGATTCGTCACC |  |
| *cyxA*-RT-F | TCGCCGTCAACTTCGGTAT | Amplification of *cyxA* gene |
| *cyxA*-RT-R | TTCATAGGTCAGCAGAGGGC |  |
| *cyxB*-RT-F | GTCTTTGCCACGCTGATGT | Amplification of *cyxB* gene |
| *cyxB*-RT-R | GCGACGCTGTTGACCATTA |  |
| *cyxA*-*cyxB*-F | tgatcgctattctcgcc | Amplification of the fragment between *cyxA* and *cyxB* genes |
| *cyxA*-*cyxB*-R | CGACGCTGTTGACCATTA |  |
| *cyxB*-KP_RS14000-F | CAGCCTGCCGAATCTCTAT | Amplification of the fragment between *cyxB* and KP_RS14000 genes |
| *cyxB*-KP_RS14000-F | TCCCACTCAGACTTTTAGCC |  |
| *katE*-A | GTATGCGGCCGCagcaggcggaagaagata | Amplification of the *katE* left flanking sequence |
| *katE*-B | AATGAAAGCAGGAGACGAGTCGGGCTTGATTCACTTTC |  |
| *katE*-C | gaaagtgaatcaagcccgACTCGTCTCCTGCTTTCATT | Amplification of the *katE* right flanking sequence |
| *katE*-D | GTATGCGGCCGCGGCTTCAATCTCACCTTCC |  |
| *katG*-A | GTATGCGGCCGCGTGTTATTTCCCGACTGGAT | Amplification of the *katG* left flanking sequence |
| *katG*-B | TTCATCACCTTGGTCCAGGTATCAGGCTCCGTTTGCT |  |
| *katG*-C | agcaaacggagcctgaTACCTGGACCAAGGTGATGAA | Amplification of the *katG* right flanking sequence |
| *katG*-D | GTATGCGGCCGCCGGTGATGGACTATCTGAAGC |  |
| *katG*-RT-F | AACTACGACGGCAGCAAA | Amplification of *katG* gene |
| *katG*-RT-R | TGGAGCCAAACACCAGAT |  |
| *katE*-RT-F | ATCGTCGGCAATGACTTC | Amplification of *katE* gene |
| *katE*-RT-R | CAATGGCGTGGAGAAAG |  |
| *recA*-RT-F | TTAAACAGGCCGAATTCCAG | Amplification of *recA* gene |
| *recA*-RT-R | CCGCTTTCTCAATCAGCTTC |  |

Note: The underlines represent restriction enzyme sites (GCGGCCGC: *Not*Ⅰ; GGGCCC: *Apa*I)
